# Prostate-Specific Antigen Bounce Following Stereotactic Body Radiation Therapy for Prostate Cancer

**DOI:** 10.3389/fonc.2014.00008

**Published:** 2014-01-28

**Authors:** Charles C. Vu, Jonathan A. Haas, Aaron E. Katz, Matthew R. Witten

**Affiliations:** ^1^Division of Radiation Oncology, Winthrop-University Hospital, New York, NY, USA; ^2^Stony Brook School of Medicine, New York, NY, USA; ^3^Department of Urology, Winthrop-University Hospital, New York, NY, USA

**Keywords:** PSA, SBRT, SAbR, prostate cancer, CyberKnife

## Abstract

**Introduction:** Prostate-specific antigen (PSA) bounce after brachytherapy has been well-documented. This phenomenon has also been identified in patients undergoing stereotactic body radiation therapy (SBRT). While the parameters that predict PSA bounce have been extensively studied in prostate brachytherapy patients, this study is the first to analyze the clinical and pathologic predictors of PSA bounce in prostate SBRT patients.

**Materials and Methods:** Our institution has maintained a prospective database of patients undergoing SBRT for prostate cancer since 2006. Our study population includes patients between May 2006 and November 2011 who have at least 18 months of follow-up. All patients were treated using the CyberKnife treatment system. The prescription dose was 35–36.25 Gy in five fractions.

**Results:** One hundred twenty patients were included in our study. Median PSA follow-up was 24 months (range 18–78 months). Thirty-four (28%) patients had a PSA bounce. The median time to PSA bounce was 9 months, and the median bounce size was 0.50 ng/mL. On univariate analysis, only younger age (*p* = 0.011) was shown to be associated with an increased incidence of PSA bounce. Other patient factors, including race, prostate size, prior treatment by hormones, and family history of prostate cancer, did not predict PSA bounces. None of the tumor characteristics studied, including Gleason score, pre-treatment PSA, T-stage, or risk classification by NCCN guidelines, were associated with increased incidence of PSA bounces. Younger age was the only statistically significant predictor of PSA bounce on multivariate analysis (OR = 0.937, *p* = 0.009).

**Conclusion:** PSA bounce, which has been reported after prostate brachytherapy, is also seen in a significant percentage of patients after CyberKnife SBRT. Close observation rather than biopsy can be considered for these patients. Younger age was the only factor that predicted PSA bounce.

## Introduction

Prostate cancer remains the most commonly diagnosed non-cutaneous cancer in men (1). Stereotactic body radiation therapy (SBRT) is an emerging modality in the treatment of low- and intermediate-risk prostate cancer, with several series showing excellent local control and toxicity (2–5).

Prostate-specific antigen (PSA) bounce after brachytherapy has been well-documented (6–9). The etiology of this phenomenon is unclear; proposed explanations include laboratory error (10), a delayed radiation effect (8), and prostatitis secondary to needle insertion (11). PSA bounce has also been identified in three-dimensional conformal radiation therapy (3D-CRT) (12) and intensity-modulated radiation therapy (IMRT) (13). There have been a limited number of studies reporting the incidence of PSA bounce after SBRT, which has varied from 17 to 31% in the literature (2–5, 14). While the clinical, pathologic, and dosimetric parameters that predict PSA bounce have been extensively studied in patients undergoing permanent seed prostate brachytherapy, to our knowledge no study has performed a similar analysis in patients undergoing SBRT. This study reports our institution’s incidence of PSA bounce in prostate SBRT, as well as performs an analysis of clinical and pathologic predictors of PSA bounce in this patient population.

## Materials and Methods

### Patient population

Our institution has maintained a prospective database of patients undergoing SBRT for prostate cancer since 2006. This study was approved by our hospital’s institutional review board. One hundred twenty patients with organ-confined prostate cancer treated between May 2006 and November 2011 with follow-up of at least 18 months were included. No patients were treated with hormones during or following treatment, but patients with prior treatment with hormones were included.

### Treatment technique

All patients were treated using the CyberKnife treatment system. The CyberKnife system consists of a 6-MV linear accelerator mounted on a robotic arm. There are two orthogonal kilovoltage X-ray imagers that provide real time image guidance and automatic correction for movement of the prostate throughout the treatment. Approximately 2 weeks before treatment planning, four fiducial seeds were placed transperineally in each patient to allow for motion tracking during treatment. The four implanted seeds were positioned with two at the prostate apex and two at the base. After allowing approximately 1 week for possible seed migration, treatment planning was performed prior to the treatment day using a CT scan (1.5-mm cuts) fused to an MRI. All pre-treatment imaging was performed with the patient in the same position used for treatment delivery. For low-risk patients, the prostate alone was the gross target volume (GTV). For intermediate- to high-risk patients, the proximal half of the seminal vesicles was added to the GTV if the Gleason Score >6 and the PSA >10 ng/mL. Following delineation of the GTV, a margin was added to create the planning target volume (PTV). For low- and intermediate-risk patients, the margin was 5 mm throughout except posteriorly by the rectum where a 3-mm margin was used. For high-risk patients, an 8-mm margin was added on the involved side. Target tracking and patient positioning were accomplished by registering the location of the fiducial markers in the real time images to their planning CT location. The robotic delivery system automatically adjusts the linear accelerator position to correct for both rotational and translational movement of the patient and prostate during treatment. The total clinical accuracy for treatment is less than 1 mm. Treatments were delivered on five consecutive days. Patients had a bowel preparation prior to treatment, including Dulcolax (Boehringer, Germany) and a fleet enema on the morning of each treatment. Additionally, patients received 1500 mg of amifostine (MedImmune, LLC, Gaithersburg, MD, USA) mixed in saline as a rectal suppository at least 15–20 min prior to each treatment as a radioprotectant. The prescription dose was 35–36.25 Gy in five fractions.

### Follow-up

Routine PSA follow-up was performed at 3-month intervals for the first 2 years, 4-month intervals for the following year, and 6-month intervals thereafter. PSA bounce was defined as a rise of 0.2 ng/mL above a patient’s PSA nadir, followed by a decline in PSA to below that nadir (6). Time to PSA bounce was calculated from the first PSA measurement that was ≥0.2 ng/mL above the patient’s nadir (8). PSA bounce size was defined from the PSA nadir to the subsequent PSA peak before falling below the prior nadir.

### Statistical analysis

Univariate analysis was performed using Pearson chi-squared tests for categorical variables and Mann–Whitney *U* tests for categorical variables. Multivariate analysis was performed using logistic regression. All statistical analysis was done in SPSS 21 (IBM, Armonk, NY, USA).

## Results

One hundred twenty patients were included in our study. Demographic, tumor, and treatment characteristics are summarized in Table 1. The average age was 68.1 years (range 47–88 years). Using NCCN guidelines, 69 (58%) patients were low-risk, 38 patients were intermediate-risk (32%), and 13 patients were high-risk. Seventy-three (61%) patients were Gleason score 6 or less, 35 (29%) patients were Gleason score 7, and 11 patients were Gleason score 8 or higher. Median prostate size was 69.9 g (range 24–201 g).

**Table 1:** **Patient and tumor characteristics**.

Age	68.1 (47–88)
Prostate size	69.9 (24–201)
Pre-Tx PSA	5.5 (1–34)
**NCCN RISK**	
Low	69 (58%)
Intermediate	38 (32%)
High	13 (11%)
**GLEASON SCORE**	
≤6	73 (61%)
7	35 (29%)
8	10 (8%)
9	1 (1%)
Unknown	1 (1%)
**DOSE**	
19.5	2 (2%)
21	4 (3%)
35	63 (53%)
36.25	51 (43%)
**T-STAGE**	
T1	102 (85%)
T2	17 (14%)
Recurrence	1 (1%)
**RACE**	
White	88 (73%)
Black	14 (12%)
Spanish	7 (6%)
Other	11 (9%)
**FAMILY HISTORY OF PROSTATE CANCER**	
Yes	27 (23%)
No	93 (78%)
**PRIOR HORMONES**	
Yes	11 (9%)
No	109 (91%)

*NCCN, National Comprehensive Cancer Network; Tx, treatment; PSA, prostate-specific antigen*.

Median PSA follow-up was 24 months (range 18–78 months). Thirty-four (28%) patients had a PSA bounce. The median time to PSA bounce was 9 months, and the median bounce size was 0.50 ng/mL. Table 2 summarizes the univariate analysis of patient and tumor characteristics on the incidence of PSA bounces. Only younger age (*p* = 0.011) was shown to be associated with an increased incidence of PSA bounce. Other patient factors, including race, prostate size, prior treatment by hormones, and family history of prostate cancer, did not predict PSA bounces. None of the tumor characteristics studied, including Gleason score, pre-treatment PSA, T-stage, or risk classification by NCCN guidelines, were associated with increased incidence of PSA bounces. On multivariate analysis, younger age was the only statistically significant predictor of PSA bounce (OR = 0.937, 95% confidence interval = 0.892–0.984, *p* = 0.009).

**Table 2:** **Univariate analysis of PSA bounces**.

Variable	*P*-value
**PATIENT**	
Age	0.011
Race	1.00
Family history	0.75
Prior hormones	0.94
Prostate size	0.46
**TUMOR**	
T-stage	0.62
Gleason score	0.19
Pre-Tx PSA	0.77
NCCN risk	0.45

*PSA, prostate-specific antigen; Tx, treatment; NCCN, National Comprehensive Cancer Network*.

## Discussion

The reported rate of PSA bounce after SBRT has varied from 17 to 31% in the literature (2–5, 14). Table 3 summarizes the results of these studies. Our hospital’s experience is in-line with other institutions’ reported frequencies of PSA bounces. With institutions reporting PSA bounces in 3D-CRT (12) and IMRT (13), the PSA bounce phenomenon is seen in external beam radiation therapy (EBRT) as well as in brachytherapy.

**Table 3:** **Review of previous prostate SBRT studies**.

Study	Number of patients	PSA bounce definition	PSA bounce frequency (%)	Median bounce size (ng/mL)	Median time to PSA bounce (months)
King et al. (4)	41	Increase of ≥0.2 ng/mL followed by decline to or below previous nadir	29	0.39	18
Lee et al. (14)	29	Increase of ≥0.2 ng/mL followed by decline to or below previous nadir	28	0.69	9
McBride et al. (5)	45	Increase of ≥0.4 ng/mL followed by decline to or below previous nadir	20	1.07	11.6
Chen et al. (2)	100	Increase of ≥0.2 ng/mL followed by decline to or below previous nadir	31	0.5	15
Katz et al. (3)	304	Increase of >0.2 ng/mL from nadir	17	0.55	30
Present study	120	Increase of ≥0.2 ng/mL followed by decline to or below previous nadir	28	0.5	9

*SBRT, stereotactic body radiation therapy; PSA, prostate-specific antigen*.

Varying definitions of PSA bounce have been used in the literature. Some have used an absolute increase (e.g., 0.4 ng/mL) from the previous PSA (9), while others have used a relative increase (e.g., 15%) from the most recent PSA (15). The rate of reported PSA bounce will increase as lower thresholds of PSA bounce are used. For example, Stock et al. (9) reported a PSA bounce rate of 31% if a 0.1 ng/mL threshold was used, while the PSA bounce rate in the same population would be 17% if a 0.4 ng/mL threshold was used.

To our knowledge, this is the first study of the clinical factors that predict PSA bounce in prostate cancer patients undergoing SBRT. In a recently published multi-institutional phase I feasibility study of 45 patients, McBride et al. found that prostate SBRT patients with PSA bounces were younger than those who did not have a PSA bounce (5). However, their study only analyzed the effect of age and not the effects of other clinical and pathologic parameters.

The predictors of PSA bounce in patients undergoing permanent seed brachytherapy has been extensively discussed and was reviewed by Caloglu and Ciezki (7). While a multitude of different clinical, pathologic, and dosimetric parameters have been identified as predictors of PSA bounce, the authors found that only younger age has consistently been associated with PSA bounce. Our study suggests that this association can be extended to prostate cancer patients undergoing SBRT.

Sheinbein and colleagues performed an analysis of clinical, pathologic, and dosimetric parameters associated with PSA bounce in 102 patients undergoing IMRT for localized prostate cancer (13). They reported a PSA bounce rate of 32.4%. Only age ≤70 was associated with PSA bounces; all other parameters studied were not found to be correlated with PSA bounces.

The reasons why younger patients more frequently experience PSA bounces are unknown. One common etiology suggested is that younger men have increased androgen levels (9, 16). Another frequently cited explanation is the increased sexual activity in younger men (16, 17). Recent sexual activity could possibly lead to spurious PSA elevations, and Das et al. reported that a common cause of PSA bounces is recent ejaculation (15). However, Crook et al. found that pre-treatment potency was a univariate predictor of PSA bounce, but this association was lost on multivariate analysis (18).

Limitations of this study include its retrospective design, small sample size, and relatively short follow-up period. It is possible that some factors did not achieve statistical significance because the study was underpowered and not enough patients were studied. Larger studies with longer follow-up are needed to determine if any other factors can predict PSA bounce in prostate SBRT patients.

## Conclusion

We report the incidence of PSA bounce in our series of prostate SBRT patients to be 28%, in-line with other institutions’ experiences. As observed in studies that used brachytherapy or other types of EBRT, we identified age as the only clinical factor that predicts PSA bounce.

## Conflict of Interest Statement

Dr. Jonathan A. Haas has received speaker’s honoraria from Accuray Inc., Sunnyvale, CA, USA. The other co-authors declare that the research was conducted in the absence of any commercial or financial relationships that could be construed as a potential conflict of interest.
